# New Surgical Frontiers for Nutrition in Children

**DOI:** 10.3390/children8050400

**Published:** 2021-05-15

**Authors:** Carmine Noviello

**Affiliations:** Pediatric Surgery Unit, Department of Woman, Child, General and Specialized Surgery, University of Campania “Luigi Vanvitelli”, 81100 Naples, Italy; carmine.noviello@libero.it

Nutrition in pediatric age, if properly adapted to the various developmental phases, can be considered the first prevention tool for the most common pathologies of this age. New diagnostic approaches and new surgical techniques have made nutrition possible even in neurologically impaired children or patients with malformations. The Special Issue “Pediatric Gastroenterology and Nutrition” underlines the importance of the nutritional status in preventing important pathologies of the infant and evaluates diagnostic and surgical techniques to obtain, in a minimally invasive way, the most suitable means to feed the patient.

Proper nutrition is the basis of regular biological development and psychological condition of the child. In fact, it is essential to satisfy the child’s nutritional needs, that is, the quantity of calories and nutrients necessary to ensure an optimal state of health and an adequate growth to their genetic potential and age [1]. The nutrition of the child, if appropriately adapted to the phase of development, can be considered a good primary prevention tool for the most common pediatric diseases. In the treatment of childhood diseases, in general, nutrition plays a fundamental role both as an exclusive therapy and as a support to pharmacological treatment. In the case of childhood neoplasms, a good nutritional status allows the child to cope better with the side effects caused by the disease itself and by the therapies and anticancer drugs given to the child. At the same time, it allows the child to meet the energy demands for the normal growth process and development. It also guarantees evident positive effects on the quality of life, on the immune status, and on the infectious risk of the child.

Pediatric food intake disorders occur in children with genetic syndromes, neurological disorders, and surgical or stomach disorders. In recent years, with the development of neonatal intensive care and neonatal resuscitation techniques, an increasingly large group of patients have survived, reporting important neurological damage that creates swallowing and food dysfunctions in general. Over the past 40 years, the prevalence of cerebral palsy has risen to well over 2.0 per 1000 births [2]. Over the same period, the proportion of low-birth-weight infants has increased, and the most disadvantaged socioeconomic population is most affected. Half of these children have gastrointestinal and feeding problems.

Gastrostomy has been an operation performed in pediatric age for more than 100 years, but in the last 40 years, the techniques have improved considerably, allowing even very young patients to be treated, who may then have an adequate nutritional supply even if they are unable to swallow and they are not taking adequate nutrients orally.

Furthermore, the improvement of diagnostic and surgical techniques allows the best results of gastrostomy even in complex patients. In this case, it is easier to diagnose gastroesophageal reflux disease or stomach fixation defects that can also be treated with minimally invasive techniques, in order to have at the same time the possibility of enteral feeding and have protection for the respiratory tract.

The Special Issue, “Pediatric Gastroenterology and Nutrition”, mainly analyzes the problem of appropriate nutrition in relation to pathologies of the pediatric age.

In the first paper, Miguel Baños-Peláezand and colleagues carried out a retrospective case-control study on premature infants and found that feeding with exclusive mother’s own milk and fortified human milk can prevent necrotizing enterocolitis compared to fasting on days 7 and 14 [3].

Francesca Destro and Gloria Pelizzo presented a very interesting study on the opportunity to use a temporary gastrojejunostomy to treat gastro-esophageal reflux disease in particular patients, such as those with long-gap esophageal atresia repair. The placement of the gastrojejunostomy is much less invasive than the fundoplication, so it is useful to postpone the definitive treatment of reflux disease in these cases [4]. Furthermore, in another paper they underline the crucial role of correct nutritional status in patients with esophageal atresia [5].

Another important topic in children is nutrition in neurologically impaired patients. Considering the need, or not, of a fundoplication to protect the respiratory tract, Elisa Zambaiti and her colleagues report the experience of a single center, in which there was no difference between gastrostomy with and without fundoplication [6].

Caruso Anna Maria, who evaluated the use of esophageal high-resolution manometry during laparoscopic fundoplication in order to reduce the side effects related to the technique, has presented a preliminary study [7].

Finally, Giovanni Parente and Mario Lima conducted a preliminary study on the treatment of nonresponsive fecal incontinence in children born with anorectal malformations. Their idea is to implant autologous adipose tissue in the anal sphincter, after a lipoaspiration from the abdominal wall, processing of the lipoaspirate with a Lipogems system, and intersphincteric injection of the adipose tissue processed through endosonographic assistance [8].

In conclusion, this Special Issue emphasizes the importance of nutrition in preventing disease and improving the clinical condition of neuropathic, malformed, or operated patients.
